# Time trade-off: one methodology, different methods

**DOI:** 10.1007/s10198-013-0508-x

**Published:** 2013-07-31

**Authors:** Arthur E. Attema, Yvette Edelaar-Peeters, Matthijs M. Versteegh, Elly A. Stolk

**Affiliations:** 1 iBMG/iMTA, Erasmus University, P.O. Box 1738, 3000 DR Rotterdam, The Netherlands; 2 Department of Medical Decision Making, Leiden University Medical Centre, Leiden, The Netherlands

**Keywords:** Time trade-off, Design, Methodology, Health state valuation, B40, I10

## Abstract

There is no scientific consensus on the optimal specification of the time trade-off (TTO) task. As a consequence, studies using TTO to value health states may share the core element of trading length of life for quality of life, but can differ considerably on many other elements. While this pluriformity in specifications advances the understanding of TTO from a methodological point of view, it also results in incomparable health state values. Health state values are applied in health technology assessments, and in that context comparability of information is desired. In this article, we discuss several alternative specifications of TTO presented in the literature. The defining elements of these specifications are identified as being either methodological, procedural or analytical in nature. Where possible, it is indicated how these elements affect health state values (i.e., upward or downward). Finally, a checklist for TTO studies is presented, which incorporates a list of choices to be made by researchers who wish to perform a TTO task. Such a checklist enables other researchers to align methodologies in order to enhance the comparability of health state values.

## Introduction

A cornerstone of economic evaluations is the quality-adjusted life year (QALY), a measure of quantity and quality of life. The QALY is designed to allow for comparison of treatments across conditions. However, empirical research shows that different valuation methods, used to generate the quality-adjustment part of the QALY, produce different results. Even when researchers have used the same technique to elicit values for the same health states, such as time trade-off (TTO), large differences in produced values have been found [1]. While some variation may be expected due to differences between respondents, large differences probably reflect that TTO tasks are conducted very differently so that methodological differences affect the outcome. Incomparability of information is a key problem for the legitimacy of decisions based on economic evaluations. Therefore, the comparability of TTO studies needs to be improved. The usual way to achieve this is by harmonizing data collection efforts. Unfortunately, there have been serious discussions about several aspects of the TTO methodology in recent years, leading to an increased variety of methodological approaches. In order to permit harmonized methods for data collection, we review central elements of the TTO with the aim to understand if, and how, results of TTO studies may be influenced by the specifications of the TTO.

The TTO method elicits preferences for health states by letting a subject imagine living a defined number of years in an imperfect health state. The subject then has to indicate the number of remaining life years in full health at which the respondent is indifferent between the longer period of impaired health and the shorter period of full health. Normalizing the value of full health to 1.0 leaves us the value of the impaired health state being represented by the ratio of the two periods, i.e., the number of years in full health divided by the number of years in the impaired heath state. There is little guidance on how researchers should proceed to determine this indifference point, and as a result they do it very differently. A recent comparison of the different variants that may be subsumed under the name of ‘time trade-off’ has revealed how incomparable these approaches can become. This led the authors to conclude that TTO studies may have little more in common than their objective to quantify a trade-off between length of life and quality of life [2].

Apart from the possibility that the TTO may be not an accurate measurement technique for health states after all, another point of view is to regard the co-existence of different TTO variants as a natural state in incremental method development. The TTO method may be considered finalized when it satisfies all of its requirements. This still seems not to be the case, which we illustrate by two prominent methodological deficits. A first problem in TTO concerns its feasibility. TTO has been introduced as an interviewer-based method, but researchers, who lack time or budget to collect TTO values using time-consuming interviews continue to look for alternatives. Furthermore, the TTO procedure is criticized for problems associated with valuation of health states that are considered to be worse than dead [3, 4]. Not surprisingly, such perceived problems with TTO gave rise to a number of efforts to improve this method. Innovations in TTO are found in all elements related to the methods: new tasks, different procedures for data collection and different analytical strategies. While decision makers require standardization, the lack of scientific agreement on the best methodology sparked innovation and, as a result, comparing TTO studies is harder than ever.

While the proliferation of different TTO specifications may be seen as a valuable and integral part of the scientific endeavor to achieve optimal valuation methods, methodological variation in TTO studies is problematic from a policy point of view. TTO studies are typically conducted in the context of health technology assessments (HTA), which aim to inform policy makers in making resource allocation decisions in health care. As variants of TTO are known to produce different values for the same states [5–7], this reduces comparability of the HTA submissions. Hence, it is relevant to strive for more standardization. Standards have been developed for cost studies. In contrast, little guidance is provided for health state valuation studies. To our knowledge, NICE is the only institute that has issued guidance on how TTO studies should be conducted [8]. Their guidance indicates that those who conduct a TTO study should follow the measurement and valuation of health (MVH) protocol developed in the UK EQ-5D valuation studies, based on the desire to enhance comparability with studies that derive utilities from the EQ-5D [9]. While other governmental institutions, health insurers and other organizations are equally dependent on comparable information, they have not issued similar guidance. A lack of scientific agreement combined with little attention for the need for standardization may be the reason for this.

Against this background, the current article has two aims. First, we aim to present the state of play in TTO and achieve an understanding of the merit of various ways in which the TTO method is applied. Second, ideas will be distilled about characteristics of TTO studies that ought to be reported in order to account for consequences of particular methodological choices in comparing TTO results. We bring these elements together in a checklist.

## Methods

To identify characteristics of TTO exercises that affect outcomes, we followed a convenient approach. Our starting point was the list proposed by Stalmeier et al. [10]. This list was expanded on the basis of expert opinion, literature and knowledge of the recent developments in TTO explored in the EQ group research program. As a result, we came up with a list of factors that investigators on TTO have to decide on before conducting a TTO elicitation (Tables 1, 2, 3). This list is not comprehensive as some aspects were deliberately excluded. For instance, paper-based TTOs were omitted, because the development of computerized designs has made this very uncommon in recent TTO elicitations.

**Table 1:** Methodological aspects

Methodological question	Specification	Not applicable/not specified
1. What value range was assessed?	□ Both states BTD and WTD	□
	□ Only states BTD	
2. What method was used for valuation of worse than dead states?	□ Classical TTO	□
	□ MVH protocol	
	□ Lead-time TTO	
	□ Lag-time TTO	
	□ Composite TTO	
	□ Other	
3. What was the disease duration?	□ 10 years	□
	□ Life time	
	□ Other	
4. Was the smallest tradable unit listed?	□ Yes	
	□ No	
5. Was the lead or lag time listed?	□ Yes	□
	□ No	
6. What iteration procedure was used?	□ Bisection	□
	□ MVH fixed sequence	
	□ Other	
7. What was the response scale?	□ Years lived in full health	□
	□ Years lived in impaired health	

In order to add structure, the identified characteristics have been subsumed under three headings: methodological issues, procedural issues and analytical issues. All these issues can be expected to influence results, based on either theoretical predictions or on empirical findings. Issues are classified as methodological if they are of a substantive nature, i.e., these issues specify how the objective of measuring the trade-off between quantity and quality of life is attained. They are classified as procedural if they are more related to the appearance and structure of the experiment. Finally, issues related to analyses of the results are classified as analytical issues.

Information about how TTO study characteristics affect values was extracted from the literature by AA and YEP. We have mainly drawn upon our extensive knowledge of this literature and previous review articles, such as Arnesen and Trommald [2] and Attema and Brouwer [11]. However, we do not claim to have covered all existing literature, since no systematic literature review was performed.

## Results

In total, 25 characteristics of TTO were derived that may influence the valuations obtained by the TTO method (Tables 1, 2, 3). The greatest variation across TTO studies has been observed in methodological and procedural aspects. The effect of procedural aspects on the obtained values is often not clear. For methodological aspects, more information is available. Below, we discuss each of these factors in turn, including the predicted effects of these factors on the results, based on both theory and empirical studies.

**Table 2:** Procedural aspects

Procedural question	Specification	Not applicable/not specified
1. What was the mode of administration?	□ Face-to-face interviews	□
	□ Group interviews	
	□ Self-administered questionnaire	
	□ Internet experiment	
	□ Other	
2. Were visual aids used?	□ Yes, TTO board and health state cards	□
	□ Yes, computer assisted	
	□ No	
3. What context effects were considered?	□ Warm-up task: TTO	□
	□ Warm-up task: other utility valuations	
	□ Starting point bias	
	□ Ordering of states	
	□ Other	
4. What was the sampling frame?	□ General population	□
	□ Patients	
	□ Health-care providers	
	□ Significant others	
	□ Other	
5. Were all health state values observed?	□ Yes (direct valuations)	□
	□ No (indirect valuations)	
*If indirect valuations were used*		
6. Which modeling approach was used?	□ Multi-attribute utility theory	□
	□ Statistical inference	
7. How many respondents were included?	□ <200	□
	□ ≥200	
	□ ≥400	
	□ ≥800	
8. Number of health states to be valued	□ <50	□
	□ >50	
9. How were health states selected? (*multiple answers possible*)	□ Covering severity range	□
	□ Orthogonality	
	□ Health state plausibility	
	□ Other	
10. Lowest health state in the valuation task	□ PITS	□
	□ Other	
11. Model fit criteria	□ Mean absolute error (MAE)	
	□ Root mean square error (RMSE)	
	□ Other	
*If direct valuations were used*		
12. How was the health state described?	□ Own health	□
	□ In generic terms	
	□ In disease-specific terms	
13. How were the health state descriptions generated? *(multiple answers possible)*	□ Extracted from an existing questionnaire	□
	□ Expert experience	
	□ Literature	
	□ Other	
14. How was the text of the description structured?	□ Narrated with label	□
	□ Narrated without label	
	□ Bulleted with label	
	□ Bulleted without label

**Table 3:** Analytical aspects

Analytical question	Specification	Not applicable/not specified
1. What exclusion criteria were used?	□ Feasibility questionnaire	□
	□ Indifference between health states	
	□ Number of iterations	
	□ Trade-off, non-traders	
	□ Logical errors, ordering errors	
2. How was the best possible health defined?	□ Absence of a disease	□
	□ Perfect health	
3. How were WTD values analyzed?	□ Transformation *x*/(1−*x*)	□
	□ Analyses of the medians	
	□ Other	
4. Was the TTO adjusted for time preference?	□ Yes, by separately eliciting time preferences	□
	□ Yes, by including multiple time frames	
	□ Yes, by using one fixed discount rate	
	□ Yes, by performing both a lead- and lag-time TTO	
	□ No	

### Methodological aspects

Table 1 summarizes methodological aspects of a TTO study, which are discussed below.

#### Value range spanned

One of the most debated characteristics of TTO methodology is the strategy for evaluation of worse than dead (WTD) states. This issue arises because the method outlined above is only able to elicit positive values, i.e., values of better than dead (BTD) states, since negative life years are obviously not possible.^1^ Hence, a separate method is needed for health states that bring negative utility, if researchers wish to estimate both BTD and WTD states.^2^

Tilling et al. [12] reviewed the literature to see how this issue was addressed. They found that valuation of WTD states is often not pursued, but when it is, most often the MVH protocol is followed. The MVH procedure uses a sorting question to find out if a respondent considers a disease state BTD or WTD: a respondent chooses between immediate death and living (*t*) years in the disease state. In case the respondent would opt for immediate death, this would indicate the respondent considered the health state [at least when lasting for (*t*) years] to be WTD. Since dead is usually assigned a value of 0, this health state should have a negative utility. Subsequently the *WTD procedure* is started, where immediate death is compared to a health profile consisting of both a period in full health and a period of equal length in the disease state to be valued [13]. If the respondent still prefers immediate death, the period in full health is lengthened and the period in the disease state shortened, and vice versa. In this way, it is possible to elicit any negative utility between zero and minus infinity; in practice, the lowest attainable value depends on the *smallest tradable unit*. When the unit is small, very negative numbers, possibly even approaching minus infinity may be observed. However, because of the conceptual and practical problems this evoked, especially the need for two different kinds of questions for BTD and WTD states, a better alternative has been called for [12]. Tilling et al. identified three alternatives: lead-time TTO, lag-time TTO and chained TTO. The *lead*-*time and lag*-*time* approaches [3] were identified as the most promising.

Adding lead (lag) time in full health to both options of the TTO allows for a uniform procedure to value BTD and WTD states, which is the key theoretical advantage of the lead- and lag-time approaches. It introduces new methodological questions, such as those related to the *length of the lead (lag) time*, the *length of the disease time*, and the ratio between the lead (lag) time and disease time. Pilot studies suggested that lead-time TTO is susceptible to a framing effect that also affects BTD values (dragging them down in comparison to values observed in classical TTO) [14–16]. This effect worsens with lengthening of the lead time relative to the disease time.

#### Time frame

The total time frame, composed of disease duration and lead or lag time, if applicable, is important because TTO values are often found to vary with it [11, 17, 18], notwithstanding the fact that the QALY model predicts them to be independent of the time frame, as implied by the condition of constant proportional trade-offs (CPTO). However, no systematic effect appears from empirical studies. A tendency for TTO values to decrease with time frame is found, but a lot of mixed evidence and even studies reporting an increasing relationship also exist, making it hard to reach definitive conclusions [17]. Most TTO studies use a 10- or 20-year time frame [2], less often actuarial life expectancy is used [18, 19], and sometimes respondents’ own life expectation is used [20–22]. A clear-cut answer to the question of which of these strategies is most appropriate does not exist, because a trade-off must be made between the desire to use a realistic time frame for a condition and minimization of distorting effects, such as loss aversion and time preference [23]. One explanation for violations of the CPTO is a positive, non-exponential, *time preference*. This brings us to the issue of whether or not, and, if so, how, to deal with time preferences, which is addressed below (see analytical issues).

#### Iteration procedure

The general opinion is that some kind of choice-based iteration process works better than directly asking for an indifference value [24]. Therefore, we only focus on this possibility in this article. Within this approach, there are several variants. One is to use a bisection procedure. A second is to add or detract one unit at each consecutive question, also known as top-down titration, as was done in the MVH study [9]. The main difference between these two variants is that the number of iterations is fixed for the former, whereas it depends on the respondent’s answers in the latter. Respondents may be aware of this property of top-down titration and try to answer strategically in order to finish the experiment sooner [12]. Next to bisection and top-down titration [25], a ping-pong approach can be used. Health state valuations are between 0.10 and 0.15 higher with titration compared to ping-pong [26]. The highest attainable value is 1.0 if non-trading is allowed, and some smaller number if non-trading is not allowed or if the iteration procedure does not allow for an indifference value of exactly 1 [16].

The iteration procedure is further characterized by its *first questions*. While variables such as these staring points may be considered irrelevant to people’s perception of health states, Samuelsen et al. [27] reported that TTO values are influenced by anchoring. Specifically, this study reported an upward shift of values with higher starting points. It is common to start with a comparison of (*t*) years in the disease state to (*t*) years in full health. Because the latter option dominates the former, it would then be rational to choose the latter. If the respondent instead chooses the former option, it may indicate s/he has not (yet) understood the question, and hence it may be a natural way to test for reliability. If the respondent chooses the dominant option, a logical follow-up question is to let the respondent choose between immediate death and (*t*) years in the disease state. This allows for a division into BTD and WTD states, although a problem with this approach is that it does not take into account the possibility of maximum endurable time states, i.e., states that give a positive utility for some period *x* < *t*, but a negative utility afterwards, such that the overall utility after (*t*) years is negative [28–31].

#### Response scale

The response scale that is applied in virtually all TTO studies is duration, because duration is a quantitative variable, and quality of life is by definition qualitative (i.e., it needs some kind of description), making it difficult to let respondents give an answer in terms of quality of life. A remaining issue is which duration to use as the response variable. Most researchers use the duration in full health for this, but it may also be the duration in the disease state. In the latter case, one may fix the duration in full health and ask for the duration in the disease state that renders indifference [6, 7, 32–35]. This generally causes TTO values to become much lower, so this is an important decision to be made. Furthermore, it is possible to choose another health state than full health as the anchor state against which to value a particular health state [36]. However, proper valuation then first requires the assignment of a value to this alternative anchor state. This can only be achieved if eventually full health is used as the anchor state, implying the use of full health as anchor state cannot be avoided. In order to prevent the additional effort of having to perform multiple tasks, as well as the expected biases resulting from chaining [37], it would therefore be advisable to directly anchor on full health.

### Procedural aspects

We now turn to a discussion of the procedural aspects that are illustrated in Table 2.

#### Mode of administration

The mode of administration will also influence the results [38]. The most preferable mode is to use personal interviews, which are the most expensive as well. The advantage is that interaction between interviewer and respondent promotes good data quality. Disadvantages are the high cost and possible interviewer effects. TTO studies have increased in size over time, often necessitating the participation of multiple interviewers. The effort that is made to minimize interviewer differences is therefore relevant (e.g., training, availability of an interview script and intervision). Moreover, the interviewer help may lead to interviewer bias such as socially desirable bias and acquiescence bias. For example, respondents find it easier to agree than to disagree [39]. Even small verbal reinforcements have been shown to lead to different reactions of respondents [40].

Internet experiments have emerged as a way to obtain large representative data sets against relatively low costs [41, 42]. However, Internet experiments do not allow the researcher to monitor the effort put forward by the respondent, nor do they give the respondent the opportunity to ask questions for clarification or feedback. Versteegh et al. [41] in this issue report that Internet studies can be problematic for eliciting TTO tariffs. In-between these two is the group experiment, where sessions with small groups are run, with one experimenter present for about each 4–10 respondents. After a plenary description of the purpose of the experiment, the respondents can then answer with the experimenters walking around and answering questions if needed. Although these studies have been shown to be feasible [43, 44], this method also seems less favorable than personal interviews.

#### Visual aids

Investigators of TTO tend to have a preference for the use of graphs/illustrations to present the choice situation, since it appears that respondents find this easier than a numerical description [45, 46]. In the old days, TTO boards were commonly used. Today, the norm is computer-assisted personal interviews, because they promote correct implementation of the iteration procedure as well as a graphical illustration of the tasks. The *visual presentation* still varies between studies, which may influence results [47]. Often, a screen-shot of valuation software or applied visual presentation is requested during the peer-review procedure for the publication of results of TTO studies.

#### Context effects

People tend to learn during a TTO experiment [48]. This is typically dealt with by inclusion of a warm-up task. TTO applications differ in the efforts put in to familiarize respondents with both the tasks and the health problems under consideration. Common *warm*-*up tasks* are TTO questions using different health states or valuation of the same health states using *different* valuation techniques (such as the visual analog scale, a ranking task, discrete choices or best-worse scaling) [41, 49, 50]. A further concern is the *order of health states* that a respondent has to value. Randomization is common practice, but more research into the most appropriate strategy may be warranted. Pinto Prades found in a recent study that the precision of health state values is contingent on *ordering of the states* [51]; more precise values are obtained when a TTO sequence begins with a mild state rather than a severe state.

#### Sampling frame

It is generally recognized that the value of a health state varies with the sampling frame. Economic evaluations in the setting of health care are recommended to be made from the social perspective. Organizations involved in developing guidelines on the use of new and existing treatments, such as the National Institute for Health and Clinical Excellence (NICE), the panel of the US Public Health Service and the Dutch Health Care Insurance Board (CvZ), prefer health state values elicited from a fully informed representative sample of members of the public [8, 52, 53]. It might be challenging to fully inform members of the public. Instead, there are good arguments to use a patient sample, because these people are more familiar with the symptoms of the disease than non-patients. The panel of the US Public Health Service already suggested that in economic evaluations in which alternative interventions are compared patients' preferences might be the better choice [52]. However, when investigating a patient sample, one should be aware that adaptation and/or strategic misrepresentation may influence valuation estimates [54]. Values shaped by adaptation typically lead to smaller effect sizes in the valuation of quality of life-enhancing treatments [55]. On the other hand, the influence of adaptation will differ between health states, and it provides valid information about the perceived severity of a health state. For instance, people might better adapt to physical impairments compared to mental diseases such as depression or skin diseases such as eczema.

#### Indirect valuation

Health state valuation methods such as TTO may be used to value the health states of a health state classification system, such as the EQ-5D, the SF-6D or disease-specific questionnaires. Most classification systems contain too many health states to value all of them, and so values are elicited only for a subset. A modeling approach is used to estimate values for all health states. Modeling may be based on multi-attribute utility theory (such as with the Health Utility Index, HUI) or statistical inference. Both approaches are built on different assumptions and come with different requirements with regard to the subset of states that have to be valued directly. In the comparison of TTO values elicited from different experiments, comparing the health state selection and modeling efforts may be relevant.

When modeling is based on statistical inference, regression analysis is applied to estimate values for all health states on the basis of the subset of state observed. The impact of regression assumptions on the predicted values is greater in the case of extrapolation (outside the range of values in the data set) than in the case of interpolation (within the range of values in the data set). Therefore, it is relevant to report the *worst health state* offered to respondents in the valuation study. Furthermore, prediction intervals and goodness of fit criteria ought to be reported.

There has been little guidance to researchers about state selection, resulting in an unclear state of play. Researchers have considered covering the severity range, orthogonality and health state plausibility, but practice varies. A further issue is how many states need to be valued. Based on theory and observations, Lamers et al. [56] suggest that a minimum number of respondents per health state is required (for TTO approximately *n* = 100) and that in principle adding more states (each assessed by 100 respondents) leads to more information, hence more precise regression estimates, than increasing the number of respondents per health state. But good results have also been obtained valuing many health states with few observations per state [57]. Bagust [58] has recently argued that state selection may be improved by adopting more criteria for state selection, such as health state relevance and direct coverage of simple increments in health. Versteegh et al. [44] argue that the statistically most interesting set of health states may not be the set of health states that occurs most often in patients and show that the inclusion of the states that occur most in patients affects modeled health state values. Whatever the selection method, the selection may still result in a number of health states that is too high to value for an individual. A common solution then is to use a *blocked design*, including only a part of the subset of health states in each individual’s questionnaire, while making sure all health states of the subset are valued by a sufficient number of respondents.

In blocking the design, a concern may be obtaining a low anchor. The worst possible health state in a classification scheme is the health state where all dimensions are at their worst possible level, in other words, having severe problems on all dimensions. This state is called the PITS state. This is state 55555 in the EQ-5D-5L system. It would be advisable to include this health state in the valuation task in order to have a lower anchor, but also in this regard practice has varied. Moreover, it is essential to list the *number of health states* that were valued (overall and per respondent) and sample size, as these characteristics may also affect the predictive quality of the regression model.

When only directly observed TTO values are used rather than modeled ones, the above concerns do not apply. Direct health-state valuations could be used when a limited number of health states have to be valued, e.g., to obtain health state values to health states presented in a Markov model. This approach generates another set of methodological concerns, e.g., related to how the disease state is described (generic or disease specific terms), narrated or bulleted, labeled or unlabeled [59]. Health state descriptions are developed based on literature, on expert experience or using classification systems such as the EQ-5D, SF-6D and HUI. Health state descriptions need be specific to ensure respondents are fully informed, but also restrictive to avoid information overload. Evidence of the impact of such choices is limited. Two studies found that the exact labeling and framing of the health description did not seem to affect respondents’ valuations [60], nor did the sparseness of an EQ-5D health state description [61].

Part of direct health-state valuations are health-state valuations of the own health. This avoids the need to describe health, since the person experiencing the health problem is also the one valuing it [59]. However, health state valuations of the own health are difficult to interpret because of the lack of clarity about the health problem, e.g., respondents tend to value their whole life including minor positive [62] and minor negative events [63]. Direct health state valuations of the own health are preferred when researchers want to incorporate the effect of adaptation, for instance, cost-effectiveness analyses of psychological interventions. Direct health state valuations of the own health are also preferred for psychological illnesses [64, 65].

### Analytical aspects

This section ends with a consideration of several analytical aspects, as shown in Table 3.

#### Exclusion criteria

Several criteria can be used to exclude respondents from the analysis. One can exclude respondents who: (1) indicated they did not understand the task on a feasibility questionnaire, (2) did not differentiate between any of the different health states, (3) used only a limited number of iterations for all health states [41], (4) did not trade off any time at all (non-traders) and (5) who rated mild health states lower than severe health states [66]. Some researchers apply criterion 4 and exclude non-traders in their analysis [67–69], but this is not common practice. An average proportion of 57 % of non-traders has been reported by Arnesen and Trommald [1]. In general, cluster effects like non-trading behavior are a direct result of the desire to derive the value of a QALY by means of a trade of life years, since for some people the value of life approaches infinity. This point of view makes exclusion of non-traders inappropriate. Also criterion 5 is argued against, as its use may result in the exclusion of up to half of the respondents, and preference reversals may just indicate uncertainty. Consequently, researchers should beware of selection bias. For instance, Arnesen and Norheim [70] report that aspects of life such as having children, friends and social esteem in many cases has a higher impact than the health problem being studied. Moreover, people with lower education levels have a higher propensity to be non-traders [21, 49]. It might be questioned if researchers should exclude non-traders and respondents who misorder health states, although some researchers argue that non-traders need to be challenged by additional questions involving smaller trade units [71].

#### Definition of anchor points

In applications of TTO, the best possible health state that gets a utility of 1 is not always explicitly defined. The state that receives a utility of 1 typically is the state where the health problems for which the value is sought are absent. When using a classification scheme, such as the EQ-5D-5L, this is the health state where all dimensions are at the best possible level (i.e., no problems on any dimension; 11111 in case of the EQ-5D-5L). To avoid lengthy health state descriptions, this health state is often termed ‘full health’ or ‘perfect health.’ Care should be taken that having no problems on any of the dimensions of the description system is not necessarily the same as being perfectly healthy. For instance, the five dimensions of the EQ-5D do not capture all possible health impairments. Hence, health state 11111 does not by definition have a utility of 1 in the sense of living without any health problems. Using absence of disease instead of perfect health in cost-utility analyses seems to make health interventions appear less costly and more effective [72], although the effect on the TTO is inconclusive [73, 74].

#### Analysis of WTD values

Whenever a TTO is used, it is important to know how the analyst handles values of states considered WTD. Because those values can theoretically become minus infinity, one of them can already heavily influence the average TTO value [75]. Applications of TTO differ in what the lowest value is that can be achieved and in how extremely negative values are handled. Where lead-time TTO explicitly defines the observed value range, the MVH approach to estimating WTD values implied that the lowest achievable value was defined by the selection of the smallest unit of time that could be traded off. In most valuation studies for EQ-5D-3L, this unit was 3 months; correspondingly, the lowest achievable value was −39. Researchers have proposed and adopted a broad range of strategies to deal with extremely negative values. Negative values in TTO are often transformed in one way or another. A common *transformation* is ‘*x*’/(1−‘*x*’), constraining WTD values to −1 [76]. Alternative strategies could be to report medians instead of means or to model the data differently, i.e., on the basis of a different economic or mathematical model [77, 78].

Lead-time TTO represents an alternative way to handle negative values: by setting the ratio between lead-time TTO and disease time, the scale of observed values is explicitly defined. For example, when both the lead time and disease time are 10 years, the ratio is 1:1. The lowest possible response, i.e., declaring immediate death to give as much utility (0) as 10 years in full health followed by 10 years in the disease state then indicates 10 × 1 + 10 × ‘*x*’ = 0, i.e., ‘*x’* = *−*1. This does resolve the issue of very negative values; however, it comes at the cost of WTD values being censured to −1. One solution to this constraint is to extend the lead time and thus to modify the ratio of lead time to disease time, enabling lower minimum WTD values. But this strategy is not expected to remove the problem. The piloting studies of lead- and lag-time TTO indicate that a significant fraction of the sample expresses preferences at the very bottom of the scale, even for high lead-time to disease-time ratios. Devlin et al. [14] therefore attempt to tackle this problem analytically by applying survival analysis to model their values. Given the variety of approaches that can be adopted, researchers should report the range of values that is explored and the analytical methods that were adopted to deal with extremely negative values.

#### Time preference

As TTO values are affected by time preferences, adjustment of observed TTO values can be considered. Investigators of TTO often just neglect time preferences, but this causes an underestimation of TTO scores [79]. Adjusting TTO valuation for the influence of time preference can be done by: (1) separately eliciting time preferences and using these estimates to correct the initial TTO values [80–85], (2) including multiple time frames in the TTO [86, 87], (3) correcting all TTO values using one fixed discount rate [88] or (4) performing both a lead-time and lag-time TTO [89]. Concerning (1), several methods exist to elicit time preferences, including riskless and risky (often certainty equivalence) methods. The issue of time preference is particularly important for the lead- and lag-time TTOs since these involve a longer horizon and hence are more susceptible to discounting.

Many researchers consider the measurement of time preference as rather problematic [15]. The required methods are often not up to the task. Therefore, although correcting for discounting is theoretically attractive, it is not very practical to do so. This stresses the importance of developing time preference elicitation methods that are more feasible [81] and to adopt a standardized time preference elicitation protocol alongside a standardized TTO protocol, at least for TTO studies that lie at the heart of HTA submissions.

## Discussion

This article has investigated and explored differences in TTO studies with the aim to increase understanding of how differences in methodology between studies may affect comparability. The overview makes clear that for most characteristics of TTO, best practices cannot be defined unambiguously. Our aim is not to produce guidance on how TTO studies ought to be conducted. Instead, our goal is to raise increased awareness and understanding of the effects of different TTO factors that imply a need for standardization. In addition, this overview may facilitate explorations into which factors are most likely to receive the broadest acceptance.

Although drafting of guidelines was not the aim of this article, exploration of differences in how studies are conducted such as presented here may be at the heart of future developments in the area of harmonization, because we may learn what works and what does not work from existing differences in TTO studies. Sometimes we have been able to identify a best practice; on other occasions we have highlighted areas of TTO where ambiguity remains about best practice. This can be used to put together an agenda for methodological research in the area of TTO.

This study has been conducted against the background of the development of a TTO health-state valuation protocol for EQ-5D-5L valuation studies. Developing a protocol serves two goals: reducing method variation across valuation studies and dealing with perceived shortcomings in previous valuation studies. Recognizing that TTO methodology is far from standardized [2] and that none of the adopted TTO approaches may count on general acceptance to be considered a standard, the aim of the research program has been to compare the benefits of innovative solutions to existing shortcomings. The developmental process of the valuation protocol for EQ-5D-5L studies reported in this issue of the journal comprised of a series of methodologically oriented studies, all with a slightly different objective. Key identified issues for TTO are the WTD estimation approach and the effect of mode of administration on data quality. While the data quality concerns are currently dealt with by offering a mix of services (interviewer training, protocols, logistic support, data quality control tools), it appeared impossible to find an unambiguous solution for assessing the values of states that are considered worse than dead. Lead-time TTO may be theoretically sound but in practice suffers from a framing effect, which makes it necessary to shape this approach on the basis of arbitrary grounds. The current protocol therefore develops a status quo that serves to promote comparability of studies for the forthcoming years, although it should not stop evaluation of alternatives.

Since Stalmeier et al. [10] published their checklist on TTO, much progress has been made in the area of health-state valuation. However, none of the methods available for health-state valuation can claim to be the widely or universally accepted method. As such, the search for alternative methods continues. One innovative approach is the use of discrete choice experiments to collect response data that can be used to derive health-state values, such as proposed by Bansback et al. [90]. This approach resembles TTO in the respect that health states are valued in a trade-off between length of life and quality of life, but iteration is avoided. Instead, choice models are applied to responses derived from discrete choices about trade-offs between length and quality of life. We support experimentation with this method and are keen to learn to what extent it can resolve problems in TTO.

This article highlighted how factors in the TTO method may affect the elicited values and therefore restrain the comparability of results from different studies. We agree with Arnesen and Trommald that the current use of the TTO should not be regarded as the use of one specific method [2], and values need to be discussed in relation to how they were assessed, as previously emphasized by Stalmeier et al. [10]. Researchers using the TTO need to be aware of these effects when comparing their results with related literature using the TTO. This is not only a task of researchers but also of peers reviewing papers and editors. However, we feel that the responsibility of the research community stretches beyond that: our conviction is that efforts need to be made to reduce practice variation in TTO studies. As this article revealed that for most characteristics of TTO best practices cannot be defined unambiguously, guidance must be developed in such a way that a balance is found between the pros and cons of the different TTO approaches.

## Conclusion

The presented literature overview highlights the need for harmonization. By listing characteristics of TTO studies that affect the obtained values, our checklist offers support to those who might eventually attempt to bring convergence into TTO study practices.

## Footnotes

1 We will term this method “classical TTO” from now on.

2 The MVH WTD procedure has never been labeled and hence will just be termed ‘MVH WTD procedure’ in this article.
